# WF-PINNs: solving forward and inverse problems of burgers equation with steep gradients using weak-form physics-informed neural networks

**DOI:** 10.1038/s41598-025-24427-4

**Published:** 2025-11-18

**Authors:** Xianke Wang, Shichao Yi, Huangliang Gu, Jing Xu, Wenjie Xu

**Affiliations:** 1 School of Science, Jiangsu University of Science and Technology, Zhenjiang, 212003 China; 2 Zhenjiang Jizhi Ship Technology Co., Ltd., Zhenjiang, 212003 China; 3 Yangzijiang Shipbuilding Group, Taizhou, 212299 China; 4 BON BNPP CONSUMER FINANCE CO., LTD, Nanjing, 210002 China; 5 School of Computer, Jiangsu University of Science and Technology, Zhenjiang, 212003 China

**Keywords:** Engineering, Mathematics and computing, Physics

## Abstract

This study tackles the numerical challenges posed by solutions with steep gradients in the Burgers equation, particularly poor stability in high-gradient regions and the ill-posedness of inverse problems in shock wave modeling. We propose a Weak-Form Physics-Informed Neural Network (WF-PINN) that fundamentally enhances both forward and inverse problem solving. Key innovations include: (i) a weak-form integral formulation of the PDE loss, which improves training stability near shocks; (ii) enforcement of an entropy condition to ensure unique and physically consistent shock capture; (iii) a dual-network architecture for inverse problems, where an auxiliary network dedicated to initial condition reconstruction is coupled with the main solver via consistency constraints. Numerical experiments show that WF-PINNs achieve significantly higher accuracy and convergence robustness compared to strong-form PINNs, accurately resolving shock locations and amplitudes while enabling precise identification of unknown initial conditions and viscosity coefficients. The framework offers a unified and generalizable approach for solving conservation laws with discontinuities.

## Introduction

The Burgers equation, as a prototypical non-linear conservation law, is widely used in modeling and analysis in fields such as fluid dynamics, acoustics, and traffic flow^1–4^, due to its ability to accurately describe the formation, propagation, and dissipation of shock waves. Although analytical solutions are often unattainable for many practical applications, numerical methods for solving partial differential equations have become a crucial area of research in scientific computing. However, the high-gradient nature of its solutions with steep gradients (e.g. shocks) poses significant challenges to conventional numerical techniques, such as the finite element method^5,6^, the finite difference method^7,8^, and the boundary element method^9,10^. These methods typically rely on mesh refinement to approximate solutions, which entails high computational costs and inherent limitations in technical advancements. Moreover, they often suffer from issues such as strong mesh dependency and poor stability^11^. Inverse problems, including initial condition reconstruction and parameter identification, further exacerbate these difficulties due to the ill-posedness of the solutions and their sensitivity to noise, placing greater demands on the robustness of the algorithms^12^.

Since their introduction in 2019 by the research team at Brown University^13^, Physics-Informed Neural Networks (PINNs) have significantly advanced the application of deep learning in solving partial differential equations and uncovering physical laws. By integrating physical principles into the learning process, PINNs overcome the limitations of traditional machine learning approaches that rely solely on data-driven and black-box modeling, thereby establishing a new paradigm for solving PDEs. For instance, Raissi et al.^14,15^ applied PINNs to solve the deterministic one-dimensional Burgers equation and addressed inverse problems constrained by two- and three-dimensional PDEs with moderate amounts of training data, successfully inferring uncertain parameters in the Navier-Stokes equations. Building on this work, Karniadakis et al.^16,17^ extended the application of PINNs to reaction-diffusion equations and Euler equations. Furthermore, Wang et al.^18^ utilized PINNs to predict discrepancies in Reynolds stress within fluid models based on the Reynolds-averaged Navier-Stokes equations, achieving promising predictive performance. However, when using conventional Physics-Informed Neural Networks (PINNs) to solve partial differential equations (PDEs), the numerical solutions typically exhibit certain inaccuracies^19^.

In recent years, a series of optimization methods related to PINNs have been developed to overcome some of these limitations, and they have been successively applied to various types of partial differential equation problems.By embedding physical equations in residual form into the loss function, a flexible solution for both forward and inverse problems has been achieved^20,21^. To further enhance the accuracy, efficiency, and applicability of Physics-Informed Neural Networks (PINNs), a series of improved frameworks have been proposed: Bayesian PINNs (B-PINNs) incorporate Bayesian inference to address uncertainty quantification in noisy data^22^; extended PINNs (XPINNs) support complex domain problems and parallel computing via spatiotemporal domain decomposition^23^; and gradient-enhanced gPINNs significantly improve solution accuracy by utilizing residual gradient information^24^. In terms of architecture design, PhyCRNet combines convolutional and recurrent structures to efficiently handle spatiotemporal PDEs^25^, while convolutional-based PICNN and PIDCNN have been successfully applied to the simulation and prediction of Darcy flow in porous media^26,27^. To address training challenges, several studies have proposed adaptive strategies, such as adaptive collocation point movement and loss weighting^28^, meta-learning loss functions^29^, neural architecture search (NAS-PINN)^30^, and multi-fidelity modeling approaches^31^. Hard-constraint methods modify the network architecture to strictly satisfy boundary conditions, avoiding the sensitivity of weight selection in soft constraints^32^. Furthermore, PINNs have demonstrated strong potential for application across various scientific and engineering domains, such as simulating composite material curing processes^33^, predicting soil consolidation^34^, and hydraulic tomography inversion [26]. For high-dimensional, stochastic, and parametrized PDEs, notable progress has been made with methods such as the physics-informed neural operator (PINO)^35^, generative pre-trained PINNs (GPT-PINN)^36^, and meta-learning for stochastic advection-diffusion-reaction systems^37^. Concurrently, several studies have focused on optimizing the training process and enhancing generalization capabilities, such as improving loss function convergence via adaptive gradient descent algorithms (AGDA)^38^, employing adaptive transfer learning (AtPINN) to handle large parameter variations^39^, and proposing a unified causal scanning strategy to address training difficulties in time-dependent PDEs^40,41^. These efforts collectively advance the development of PINNs in computational modeling and engineering applications.

The advancement of Physics-Informed Neural Networks (PINNs) has been facilitated by the widespread adoption of automatic differentiation technology^42^ in deep neural networks. This technique allows the constraints of differential operators from partial differential equations to be incorporated directly into the design of the neural network’s loss function, thereby forming a neural network regularized by physical model constraints and transforming the PDE solving process into an optimization problem. In particular, PINNs confine the predictions of neural networks within the bounds of physical laws, liberating machine learning methods from their fundamental reliance on experimental or simulated data. This not only improves accuracy but also significantly enhances the interpretability of the model. As a mesh-free approach, PINNs circumvent the complexities associated with traditional numerical discretization and demonstrate strong generalization capabilities. They have been successfully applied across various domains, including fluid mechanics^43,44^, materials science^45–47^, and heat transfer analysis^48^. Especially in data-scarce scenarios, the integration of physical constraints compensates for insufficient data, markedly improving feasibility and robustness. However, conventional strong-form PINNs often exhibit significant limitations when handling solutions with steep gradients-such as shock waves and discontinuities-including difficulties in training convergence and blurred shock capture^49^. There is a pressing need to integrate mathematical theory, such as weak formulations, to enhance the physical consistency of PINNs in these challenging contexts.

To address the aforementioned challenges, weak solution theory provides a rigorous mathematical framework for solutions with steep gradients of conservation laws. By defining solutions in an integral form, it circumvents the reliance on solution smoothness required by direct differential equation solving^50^. Several studies have attempted to incorporate the concept of weak solutions into deep learning, such as imposing entropy conditions to constrain the network’s output^51^. However, existing approaches have primarily focused on forward problems, and the application of weak solution theory has largely been confined to a posteriori error analysis, failing to fully exploit its potential for globally guiding network training.

Furthermore, for inverse problems, a wide array of sophisticated techniques has been developed for parameter identification and model inversion in dynamical systems, which informs our approach to the inverse problems of the Burgers equation. Sensitivity-based methods offer a powerful framework for estimating unknown parameters by leveraging the gradient information of system responses^52,53^. These are particularly effective for systems where derivative information can be calculated efficiently. Parallelly, nonlinear subspace identification techniques provide robust data-driven tools for extracting reduced-order models from input-output data, even for complex structures like airfoil-store systems with inherent nonlinearities^54,55^. In a different paradigm, sparse identification of nonlinear dynamics (SINDy) has emerged as a potent method for discovering parsimonious governing equations from data^56^, though its application to strongly nonlinear systems requires careful consideration^57^. While these methods are highly successful in their respective domains, key issues remain open-such as how weak solution constraints can mitigate noise interference and how to effectively balance the selection of regularization parameters. Although the viscous Burgers equation admits smooth solutions, the small viscosity in our problem leads to the development of extremely steep gradients in the solution as time evolves. These sharp fronts pose significant challenges for numerical methods, including standard PINNs, much like true discontinuities do. To address these challenges, this paper proposes a novel weak-form theory-informed PINN framework, designed to unify the solution of both forward and inverse problems involving shock waves in the Burgers equation. The main contributions are summarized as follows: (1) Theoretical aspect: The integral form of the weak solution is embedded as a hard constraint within the PINN loss function, mathematically ensuring the convergence of the network solution in Sobolev space and overcoming the training instability of conventional PINNs caused by point-wise residual optimization in shock regions.( 2) Algorithmic aspect: An adaptive residual point sampling strategy is designed to dynamically enhance the training weight in regions near shocks. Combined with sensitivity analysis, this enables automated selection of regularization parameters, reducing the dependence of inverse problem solving on manual hyperparameter tuning. (3) Application aspect: High-precision capture of shock location and amplitude is achieved in forward problems without localized mesh refinement, while significant improvements in the robustness of parameter identification are demonstrated in noisy inverse problems. This provides an efficient and practical tool for engineering applications.

The remainder of this paper is structured as follows: Section “Problem formulation” introduces the solution with steep gradients of the Burgers equation and the foundational theory of weak solutions. Section “Methodology” details the basic framework of physics-informed neural networks (PINNs), along with the physics-informed neural networks for embedding weak-form (WF-PINNs) constraints and the design of regularization strategies for inverse problems. In “Numerical experiments”, numerical experiments are conducted to validate the effectiveness of the proposed approach, accompanied by comparative analysis with conventional methods. Finally, “Conclusion” summarizes the key findings of this study and outlines potential directions for future research.

## Problem formulation

### The burgers equation and characteristics of solutions with steep gradients

The Burgers equation serves as a fundamental model in fluid mechanics for describing nonlinear wave phenomena. Its shock wave solutions reflect the near-discontinuous characteristics of the equation under specific conditions, manifesting as physical phenomena where field variables (e.g., velocity, density) undergo abrupt jumps at certain spatial locations. The discussion below will elaborate from both theoretical and physical perspectives:

Fundamental Form of the Burgers Equation:

1 $$\begin{aligned} \frac{\partial u}{\partial t}+u\frac{\partial u}{\partial x}=\nu \frac{\partial ^2u}{\partial x^2},\quad (x,t)\in \Omega \times (0,T] \end{aligned}$$

where $\nu > 0$ denotes the viscosity coefficient. The viscous term on the right-hand side suppresses the singularity of the solution through its dissipative effect, thereby ensuring global smoothness of the solution and inhibiting shock formation. However, as the viscosity coefficient $\nu \rightarrow 0$, this form describes nonlinear wave propagation in the absence of dissipation. Under such conditions, the system loses its dissipative mechanism, and the nonlinearity-dominated wave steepening effect leads to the spontaneous formation of shocks within finite time. This is characterized by the divergence of spatial derivatives at the point of discontinuity.

Mechanisms of Shock Formation and Mathematical Reformulation:

1. Nonlinear Advective Instability: In the inviscid case, the characteristic propagation speed is positively correlated with wave amplitude. As a result, wave peaks (high-amplitude regions) propagate significantly faster than wave troughs, leading to typical wavefront compression. When characteristic lines intersect, the existence of a classical solution breaks down, mathematically corresponding to the formation of a singularity where the gradient tends toward infinity.
2. Failure of Classical Solutions: Classical solutions of differential equations require global differentiability of the solution function, which fundamentally conflicts with the physical reality of nonexistent derivatives at shock discontinuities. To overcome this theoretical limitation, the concept of weak solutions is introduced-redefining well-posedness through an integral formulation, thereby allowing physically realistic solutions containing discontinuities to be accommodated within a rigorous mathematical framework.

### Formulation of the weak solution theoretical model

The core idea of weak solution theory lies in converting the strong form of a differential equation into a weak formulation expressed in integral form, thereby relaxing the smoothness requirements on the solution and allowing it to satisfy the equation in a distributional sense. A test function $\phi (x, t)$ serves as a “detector”that transforms the differential equation into an integral equation, thereby permitting discontinuities in the solution *u*(*x*, *t*). Taking the viscous Burgers equation given in Equation (1) as an example.

**Multiply the Original Equation by a Test Function and Integrate**: For any smooth, compactly supported test function $\phi (x, t) \in C_0^\infty (\mathbb {R} \times [0, \infty ))$, multiply both sides of the equation by $\phi (x, t)$ and integrate over the entire spacetime domain:

2 $$\begin{aligned} \int _{0}^{\infty }{\int _{-\infty }^{\infty }{(\frac{\partial u}{\partial t}+u\frac{\partial u}{\partial x}-v\frac{{{\partial }^{2}}u}{\partial {{x}^{2}}}}})\phi \,dxdt=0. \end{aligned}$$

**Treat Derivative Terms Using Integration by Three Parts**: Apply integration by parts to transfer the derivatives onto the test function, thereby avoiding direct differentiation of *u*.

**1) Time Derivative Term**:

3 $$\begin{aligned} \int _0^\infty \int _{-\infty }^\infty \frac{\partial u}{\partial t}\phi \mathrm {~}dxdt=-\int _0^\infty \int _{-\infty }^\infty u\frac{\partial \phi }{\partial t}dxdt. \end{aligned}$$

For the time integral, applying integration by parts yields:

4 $$\begin{aligned} \int _0^\infty \frac{\partial u}{\partial t}\phi \mathrm {~}dt=u\phi |_{t=0}^{t=\infty }-\int _0^\infty u\frac{\partial \phi }{\partial t}dt.\end{aligned}$$

Since $\phi$ vanishes as $t \rightarrow \infty$ (due to compact support), and the boundary term at the initial time $t = 0$ is $-\int _{-\infty }^{\infty } u(x, 0)\phi (x, 0) dx$, it should be noted that the weak solution definition does not typically incorporate initial conditions explicitly (they must be handled separately). Here, the result of the time derivative term after integration is retained directly.

**2) Advection Term**:

5 $$\begin{aligned} \int _0^\infty \int _{-\infty }^\infty u\frac{\partial u}{\partial x}\phi dxdt=\int _0^\infty \int _{-\infty }^\infty \frac{\partial }{\partial x}(\frac{u^2}{2})\cdot \phi dxdt=-\int _0^\infty \int _{-\infty }^\infty \frac{u^2}{2}\cdot \frac{\partial \phi }{\partial x}dxdt. \end{aligned}$$

For the spatial integral, integration by parts gives:

6 $$\begin{aligned} \int _{-\infty }^{\infty }{\frac{\partial }{\partial x}(\frac{{{u}^{2}}}{2})\phi \,dx}=\frac{{{u}^{2}}}{2}\phi \,|_{t=-\infty }^{t=\infty }-\int _{-\infty }^{\infty }{\frac{{{u}^{2}}}{2}\frac{\partial \phi }{\partial x}\,dx}. \end{aligned}$$

Since $\phi$ vanishes as $|x| \rightarrow \infty$, the boundary term disappears.

**3) Viscous Term**:

7 $$\begin{aligned} -v\int _{0}^{\infty }{\int _{-\infty }^{\infty }{\frac{{{\partial }^{2}}u}{\partial {{x}^{2}}}\phi \,dxdt}}=v\int _{0}^{\infty }{\int _{-\infty }^{\infty }{\frac{\partial u}{\partial x}\,\frac{\partial \phi }{\partial x}\,dxdt}}-v\frac{\partial u}{\partial x}\cdot \phi |_{x=-\infty }^{x=\infty }.\end{aligned}$$

Similarly, the boundary term vanishes, and the final result is $v\int _{0}^{\infty } \int _{-\infty }^{\infty } \frac{\partial u}{\partial x} \frac{\partial \phi }{\partial x} dx dt$.

**Combine All Terms** : Substitute all integration-by-parts results into the original integral equation:

8 $$\begin{aligned} \int _{0}^{\infty }{\int _{-\infty }^{\infty }{u\frac{\partial \phi }{\partial t}\,dxdt}} +\int _{0}^{\infty }{\int _{-\infty }^{\infty }{\frac{{{u}^{2}}}{2}\cdot \frac{\partial \phi }{\partial x}\,dx}}dt =v\int _{0}^{\infty }{\int _{-\infty }^{\infty }{\frac{\partial u}{\partial x}\,\frac{\partial \phi }{\partial x}\,dxdt}}. \end{aligned}$$

After simplification of the notation, the weak solution is defined as follows: specifically, if there exists an element $u\in {{L}^{2}}(\Omega )$ in the function space such that:

9 $$\begin{aligned} \iint {\left[ u\frac{\partial \phi }{\partial t}+\frac{{{u}^{2}}}{2}\frac{\partial \phi }{\partial x}-v\frac{\partial u}{\partial x}\,\frac{\partial \phi }{\partial x} \right] }dxdt=0,\quad \forall \phi \in C_{0}^{\infty } \end{aligned}$$

where the test function $\phi$ is a smooth compactly supported function, and $C_{0}^{\infty }$ denotes the space of infinitely differentiable functions that vanish outside a bounded domain, then *u* is called a weak solution of the equation. Based on the concept of the distributional derivative, this formulation transfers the differentiation operation to the test function through an integral averaging effect, which reduces the sensitivity to local discontinuities. As a result, the solution *u* is allowed to contain discontinuities in the sense of Lebesgue integrability.

Existence and Uniqueness of Weak Solutions:

1. Existence Theorem: For conservation laws, such as the Burgers equation, the existence of weak solutions under suitable initial conditions can be established via methods such as compensated compactness or the vanishing viscosity method.
2. Uniqueness Issue:Weak solutions may not be unique. To single out the physically relevant solution, an entropy condition must be imposed. The entropy condition requires that:
  10 $$\begin{aligned} \eta {{(u)}_{t}}+\varphi {{(u)}_{x}}\le 0 \end{aligned}$$
  Here, $\eta$ denotes a convex entropy function and $\varphi$ the corresponding entropy flux, ensuring that shocks satisfy the entropy increase principle.

## Methodology

This chapter presents the Physics-Informed Neural Networks (PINNs) and the Weak-Form Physics-Informed Neural Networks (WF-PINNs) methods, and introduces how they are used to solve the steep-gradient Burgers equation.

### Physics-informed neural networks (PINNs)

**Figure 1:**
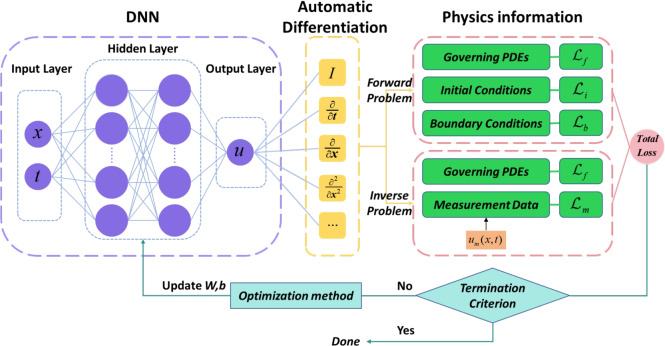
A typical PINN framework for solving partial differential equations.

Physics-Informed Neural Networks (PINNs) represent a deep learning framework that integrates physical principles. The core idea involves incorporating the residual of the partial differential equation system into the loss function, thereby embedding physical conservation laws as soft constraints to guide the learning process of the neural network. The model architecture primarily comprises two key technical components: deep neural networks and automatic differentiation. Its deep network structure forms a nonlinear mapping between the input and output layers through multiple hidden layers. The forward propagation process utilizes automatic differentiation based on the chain rule for gradient computation, while network parameters are iteratively optimized via backpropagation. To present the logic more clearly, a typical PINN framework for solving both forward and inverse PDE problems is illustrated in Fig. 1.

A fully connected feedforward deep neural network (DNN) is employed to compute the predicted value ${{u}_{pred}}({{x}_{i}},{{t}_{i}};w,b)$, which serves as an approximation to the exact value ${{u}_{exact}}({{x}_{i}},{{t}_{i}})$ at the input point $({{x}_{i}},{{t}_{i}})$. Subsequently, by minimizing the total loss function ${{\mathscr {L}}_{total}}$ and utilizing an optimization algorithm, the parameters of the DNN are iteratively trained and updated, driving ${{u}_{pred}}({{x}_{i}},{{t}_{i}};w,b)$ to converge toward the exact value ${{u}_{exact}}({{x}_{i}},{{t}_{i}})$.

In the conventional PINN framework, the input (*x*, *t*) represents the coordinates of training points, typically composed of three subsets from $T=\{{{T}_{f}}\times {{T}_{b}}\times {{T}_{i}}\}$, where ${{T}_{f}}$ denotes points sampled within the spatiotemporal domain, ${{T}_{b}}$ represents points taken on the boundary, and ${{T}_{i}}$ corresponds to points extracted from the initial condition.

11 $$\begin{aligned} \begin{aligned}&T_{f} = \{(x,t) \mid x \in [0,l], t \in [0,T]\} \\&T_{b} = \{(x,t) \mid x \in \Gamma , t \in [0,T]\} \\&T_{i} = \{(x,t) \mid x \in [0,l], t = 0\} \end{aligned} \end{aligned}$$

The loss function is designed based on the sampled points, taking into account the initial conditions described in the equation and the given boundary conditions. It integrates the equation residual, boundary conditions, and initial conditions via a weighted mean squared error, and can be defined as follows:

12 $$\begin{aligned} \mathscr {L}(w;b;T)={{\lambda }_{f}}{{\mathscr {L}}_{f}}(w;b;{{T}_{f}})+{{\lambda }_{i}}{{\mathscr {L}}_{i}}(w;b;{{T}_{i}})+{{\lambda }_{b}}{{\mathscr {L}}_{b}}(w;b;{{T}_{b}}) \end{aligned}$$

where ${{\mathscr {L}}_{f}}(w;b;{{T}_{f}})$ denotes the PDE residual loss term, ${{\mathscr {L}}_{i}}(w;b;{{T}_{i}})$ represents the initial condition loss term, ${{\mathscr {L}}_{b}}(w;b;{{T}_{b}})$ corresponds to the boundary condition loss term, and ${{\lambda }_{f}},{{\lambda }_{i}},{{\lambda }_{b}}$ are the corresponding weighting coefficients.

13 $$\begin{aligned} \begin{aligned}&\mathscr {L}_{f}(w;b;T_{f}) = \frac{1}{|T_{f}|} \sum _{(x_{i},t_{i}) \in T_{f}} \left| \frac{\partial u^{*}}{\partial t_{i}} + u^{*} \frac{\partial u^{*}}{\partial x_{i}} - v \frac{\partial ^{2} u^{*}}{\partial x_{i}^{2}} \right| ^{2} \\&\mathscr {L}_{i}(w;b;T_{i}) = \frac{1}{|T_{i}|} \sum _{(x_{i},0) \in T_{i}} \left| u^{*}(x_{i},0) - u_{0}(x_{i}) \right| ^{2} \\&\mathscr {L}_{b}(w;b;T_{b}) = \frac{1}{|T_{b}|} \sum _{(x_{i},t_{i}) \in T_{b}} \left| u^{*}(x_{i},t_{i}) - u_{boundary}(x_{i},t_{i}) \right| ^{2} \end{aligned} \end{aligned}$$

Furthermore, to address inverse problems of partial differential equations (PDEs), i.e., discovering unknown parameters within PDEs using measurement data, only minor modifications to the aforementioned forward problem setup are required. In this context, the unknown parameters are treated as hyperparameters and optimized simultaneously with the neural network. During the solution of inverse PDE problems, it is often challenging to obtain precise initial and boundary conditions. In most cases, only the governing equations and measurement data are available. Under such circumstances, the physics-informed components consist of two parts: the residual of the governing equations and the residual of the measurement data. The loss function can thus be expressed as:

14 $$\begin{aligned} \mathscr {L}(w;b;T)={{\lambda }_{f}}{{\mathscr {L}}_{f}}(w;b;{{T}_{f}})+{{\lambda }_{m}}{{\mathscr {L}}_{m}}(w;b;{{T}_{m}}) \end{aligned}$$

where ${{\mathscr {L}}_{f}}(w;b;{{T}_{f}})$ denotes the PDE residual loss term, ${{\mathscr {L}}_{m}}(w;b;{{T}_{m}})$ represents the measurement data loss term, and the coefficients ${{\lambda }_{f}},{{\lambda }_{m}}$ are their corresponding weighting coefficients, respectively.

15 $$\begin{aligned} \begin{aligned}&\mathscr {L}_{f}(w;b;T_{f}) = \frac{1}{|T_{f}|} \sum _{(x_{i},t_{i}) \in T_{f}} \left| \frac{\partial u^{*}}{\partial t_{i}} + u^{*} \frac{\partial u^{*}}{\partial x_{i}} - v \frac{\partial ^{2} u^{*}}{\partial x_{i}^{2}} \right| ^{2} \\&\mathscr {L}_{m}(w;b;T_{m}) = \frac{1}{|T_{m}|} \sum _{(x_{i},t_{i}) \in T_{m}} \left| u^{*}(x_{i},t_{i}) - u_{m}(x_{i},t_{i}) \right| ^{2} \end{aligned} \end{aligned}$$

### WF-PINNs: a neural network solver for burgers equation with solutions with steep gradients

To address the challenges of gradient explosion and solution oscillations encountered by conventional Physics-Informed Neural Networks (PINNs) in handling steep-gradient problems such as shock waves in the Burgers equation, WF-PINNs innovatively introduces the weak-form integral equation and entropy conditions as additional constraint terms into the loss function. By incorporating more powerful and physically meaningful constraints, the neural network can learn a more comprehensive set of underlying PDE rules, achieve a more accurate approximation of the solution, and significantly improve both stability and accuracy in solving the equation.

Traditional PINNs directly rely on the strong form of the PDE (e.g., involving second-order derivative terms), which is highly sensitive to derivative calculations near discontinuities. In contrast, WF-PINNs defines the loss function in an integral form by combining the original equation with test functions, thereby relaxing the smoothness requirements on the solution and effectively capturing steep-gradient phenomena such as shock waves. By transforming the equation into an integral form, WF-PINNs avoids direct derivative computation. The weak-form integral formulation of the Burgers equation is shown in Equation (9).

To effectively enhance the accuracy of shock wave capture, select physically meaningful solutions (while avoiding non-physical artifacts such as post-shock oscillations), improve solution stability in regions with steep gradients. According to the requirement of Equation (10), an appropriate entropy function $\mu (u)$ and entropy flux $\varphi (u)$ shall be introduced.In the implementation, violations of the entropy condition are penalized using the ReLU function.

**Figure 2:**
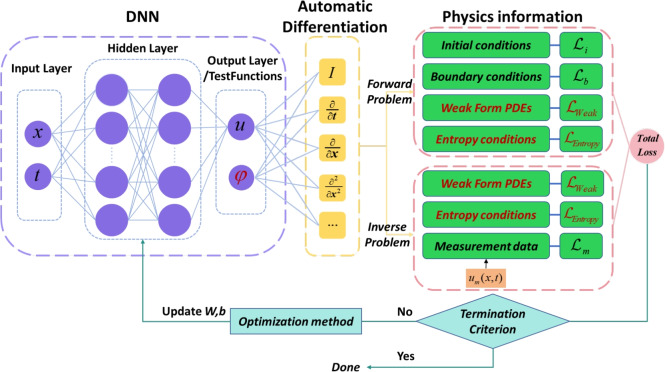
A novel WF-PINNs framework for solving partial differential equations.

The overall framework of WF-PINNs is illustrated in Fig. 2. As clearly shown in the figure, unlike the conventional PINN framework, WF-PINNs employs weak-form integral equations-primarily based on multiple smooth compactly supported test functions-to replace the residual terms derived from the classical governing equations. Simultaneously, the main and auxiliary outputs of the integration involving variables from the governing equations are computed. Furthermore, the accuracy of the solution is enhanced by enforcing an entropy condition consistent with the laws of physics. The red-marked components in the figure indicate the improved or newly introduced elements in the proposed WF-PINNs framework compared to traditional PINNs, namely the weak residual loss term and the entropy condition loss term.

Taking the forward problem of solving PDEs as an example, the loss function design of WF-PINNs generally consists of the following components: the weak residual loss, the initial/boundary condition loss, and the entropy condition loss.

16 $$\begin{aligned} \mathscr {L} = \lambda _w \mathscr {L}_{\text {Weak}} + \lambda _e \mathscr {L}_{\text {Entropy}} + \lambda _i \mathscr {L}_{\text {IC}} + \lambda _b \mathscr {L}_{\text {BC}} \end{aligned}$$

The treatment of initial and boundary condition losses remains consistent with traditional PINNs and will not be reiterated here. The weak residual loss ${{\mathscr {L}}_{Weak}}$, also referred to as the weak-form integral equation loss, is approximated via Monte Carlo integration based on a set of sampled test functions $\{{{\phi }_{i}}\}_{i=1}^{N}$. This term evaluates the integral residual as follows:

17 $$\begin{aligned} {\mathscr {L}_{\text {Weak}}}=\frac{1}{N}\sum \limits _{i=1}^{N}{{{\left| \iint {_{\Omega }({{u}_{\theta }}\frac{\partial {{\phi }_{i}}}{\partial t}+\frac{{{u}_{\theta }}^{2}}{2}\frac{\partial {{\phi }_{i}}}{\partial x}-v{{u}_{\theta }}\frac{\partial {{u}_{i}}}{\partial x}\frac{\partial {{\phi }_{i}}}{\partial x}})dxdt \right| }^{2}}} \end{aligned}$$

Additionally, an entropy condition residual term ${\mathscr {L}_{Entropy}}$ is incorporated into the loss function to ensure the network satisfies the entropy condition:

18 $$\begin{aligned} {\mathscr {L}_{Entropy}}=\sum \limits _{j=1}^{M}{\left| \max (0,\eta {{({{u}_{\theta }})}_{t}}+\varphi {{({{u}_{\theta }})}_{x}} \right| }\end{aligned}$$

When addressing inverse problems, it may be difficult to obtain exact initial and boundary conditions for the partial differential equation. In most cases, the available information may only consist of the governing equations and sparse measurement data. Of course, if additional information-such as boundary or initial conditions-is available, it can be readily incorporated into the loss function, similar to the conventional PINNs framework for solving forward problems. Here, we first consider the scenario with the least available information, i.e., only the governing equations and measurement data are known. In this case, the loss function for solving the partial differential equation within the WF-PINNs framework can be formulated as follows:

19 $$\begin{aligned} \mathscr {L} = \lambda _w \mathscr {L}_{\text {Weak}} + \lambda _e \mathscr {L}_{\text {Entropy}} + \lambda _m \mathscr {L}_{\text {m}} \end{aligned}$$

where ${{\mathscr {L}}_{Weak}}$ denotes the weak residual loss term, ${{\mathscr {L}}_{Entropy}}$ represents the entropy condition residual term, and ${{\mathscr {L}}_{m}}$ corresponds to the measurement data loss term. The coefficients ${{\lambda }_{w}}, {{\lambda }_{e}}, {{\lambda }_{m}}$ preceding these terms are their respective weighting factors.

## Numerical experiments

In this section, we present numerical examples involving solutions with steep gradients of the Burgers equation to demonstrate the performance advantages of our proposed WF-PINNs over conventional PINNs in solving both forward and inverse problems.

All code in this work is implemented based on PyTorch, a widely adopted deep learning framework in the field of machine learning. For all numerical examples in this study, the activation function is chosen as Tanh, a differentiable nonlinear activation function. The Adam optimizer is employed with a fixed learning rate of 0.001, further supplemented by a learning rate scheduling strategy to enhance training stability. Network parameters are initialized following the Xavier scheme, which effectively mitigates vanishing or exploding gradient issues by maintaining consistent signal variance throughout forward and backward propagation.

Regarding the neural network architecture, to ensure a fair comparison, identical network architectures were uniformly adopted for all methods. Furthermore, detailed information concerning the network configuration, optimizer settings, training dataset, and validation set is provided in Table 1. Here, FPs and IPs denote the forward problems and inverse problems, respectively. Depth and Width refer to the fundamental architectural configuration of the neural network, while LR represents the learning rate. $N_{\text {ic}}$, $N_{\text {bc}}$ and $N_{\text {colloc}}$ denote the sizes of the datasets for the initial conditions, boundary conditions, and PDE residual collocation points, respectively. $N_{\text {obs\_ic}}$ and $N_{\text {obs\_inter}}$ refer to the sizes of the sparse observation datasets for the initial states and interior measurement points in the inverse problem, respectively. $N_{\text {val}}$ indicates the size of the final testing dataset. The symbol“-”denotes absence or not applicable.

**Table 1:** Data configuration of PINNs and WF-PINNs for forward and inverse problems.

Type	Method	Depth	Width	LR	$N_{\text {ic}}$	$N_{\text {bc}}$	$N_{\text {colloc}}$	$N_{\text {obs\_ic}}$	$N_{\text {obs\_inter}}$	$N_{\text {val}}$
FPs	PINNs	4	64	0.001	10000	10000	10000	-	-	2000
	WF-PINNs	4	64	0.001	10000	10000	10000	-	-	2000
IPs	PINNs	4	64	0.001	–	1000	10000	100	500	2000
	WF-PINNs	4	64	0.001	–	1000	10000	100	500	2000

The network employed for solving this partial differential equation comprises 2 input dimensions (space *x* and time *t*), 1 output dimension (physical quantity *u*), and 3 hidden layers with 64 neurons each. During training, a random sampling strategy is adopted to generate collocation points, as well as boundary and initial condition data. Based on these data, the residuals of the physical constraints are computed using automatic differentiation. Gradient calculations are performed via PyTorch’s backpropagation mechanism, and gradient clipping is applied to enhance training stability. Throughout the training process, an entropy residual term and a dynamic weight adjustment strategy are specifically incorporated to improve the capability of capturing singular behaviors such as shock waves. For inverse problems, an additional sub-network dedicated to inverting the initial conditions is introduced and synergistically optimized with the main network through consistency constraints.

To assess the accuracy of WF-PINNs quantitatively, this study utilizes two key metrics: the Mean Squared Error (MSE) and the relative ${{L}_{2}}\text {-}error$. These indicators play a crucial role in measuring deviations between the model’s predicted solutions and the corresponding reference numerical solutions2. Mathematically, MSE is formulated as the average of squared residuals between predicted and true values, given by $\frac{1}{N}{{\sum \limits _{i=1}^{N}{({{u}_{i}}-\widehat{{{u}_{i}}})^{2}}}}$, where $\widehat{{{u}_{i}}}$ represents the predicted value and ${{u}_{i}}$ denotes the ground truth solution. The relative ${{L}_{2}}\text {-}error$ is defined as $\frac{{{\left\| u-\widehat{u} \right\| }_{2}}}{{{\left\| u \right\| }_{2}}}$, in which ${{\left\| \centerdot \right\| }_{2}}$ signifies the ${{L}_{2}}$ norm.

### Forward problem of the burgers equation

In this section, we address the forward problem for a hyperbolic conservation law, specifically considering the Burgers equation defined as follows:

20 $$\begin{aligned} \begin{aligned}&\frac{\partial u}{\partial t}+u\frac{\partial u}{\partial x}=\frac{0.01}{\pi }\frac{{{\partial }^{2}}u}{\partial {{x}^{2}}},\quad (x,t)\in [-1,1]\times [0,1], \\&u(x,0)=-\sin (\pi x), \\&u(-1,t)=u(1,t)=0. \end{aligned} \end{aligned}$$

Unlike equations with smooth solutions, the solution of this Burgers equation exhibits sharp interior layers, making it difficult to obtain an analytical solution. Therefore, a high-precision finite difference scheme is employed to solve the equation and obtain a reference numerical solution. Subsequently, boundary conditions and initial values are selected as the training set. Based on the known source term and governing equation, the shared parameters in the neural network are learned by minimizing the mean squared error loss. Both WF-PINNs and standard PINNs are applied to solve the aforementioned Burgers equation, and a comparative analysis is conducted to evaluate their effectiveness in capturing the sharp interior layer characteristics of the solution.

Specifically, in this scenario, the loss function of WF-PINNs is designed as follows:

21 $$\begin{aligned} \begin{aligned} \mathscr {L}&= \lambda _w \mathscr {L}_{\text {Weak}} + \lambda _e \mathscr {L}_{\text {Entropy}} + \lambda _i \mathscr {L}_{\text {IC}} + \lambda _b \mathscr {L}_{\text {BC}} \\&= \lambda _w \frac{1}{N}\sum _{i=1}^{N} \left| \iint _{\Omega } \left( u_{\theta } \frac{\partial \phi _i}{\partial t} + \frac{u_{\theta }^2}{2} \frac{\partial \phi _i}{\partial x} - u_{\theta } \frac{0.01}{\pi } \frac{\partial u_i}{\partial x} \frac{\partial \phi _i}{\partial x} \right) dxdt \right| ^2 \\ &+ \lambda _e \sum _{j=1}^{M} \left| \max \left( 0, \eta (u_{\theta })_t + \varphi (u_{\theta })_x \right) \right| \\ &+ \lambda _i \frac{1}{|T_i|} \sum _{(x_i,0) \in T_i} \left| u^{*}(x_i,0) + \sin (\pi x_i) \right| ^2 \\ &+ \lambda _b \frac{1}{|T_b|} \sum _{(x_i,t_i) \in T_b} \left( u^{*}(\pm 1,t_i) \right) ^2 \end{aligned} \end{aligned}$$

In this formulation, Gaussian test functions ${{\phi }_{i}}$ are employed to cover the entire spatiotemporal domain, mitigating local errors through weighted integration. To further select the physically relevant solution (i.e., the unique solution) near shock regions and enhance stability in these critical areas, an entropy condition is introduced, defined by the entropy function $\eta (u)=\frac{1}{2}{{u}^{2}}$ and the entropy flux $\varphi (u)=\frac{1}{3}{{u}^{3}}$. This condition is enforced by penalizing positive residuals while allowing negative residuals (which satisfy the entropy condition), thereby effectively filtering admissible solutions.

Regarding the weighting of the four loss terms in WF-PINNs, this study sets ${{\lambda }_{w}} = 10$, ${{\lambda }_{i}} = 1$, and ${{\lambda }_{b}} = 1$ to emphasize the contribution of the weak residual loss term. Furthermore, to prevent the entropy condition from dominating the optimization direction too early-which could lead to convergence to a suboptimal local solution-the training process prioritizes the physical residuals in the initial stages and gradually strengthens the entropy constraint in later phases. To achieve this, ${{\lambda }_{e}}$ is dynamically adjusted according to a piecewise linear function. The specific variation in weights can be expressed as follows:

22 $$\begin{aligned} \lambda _e = {\left\{ \begin{array}{ll} \dfrac{3e}{E}, & \text {if } 0 \le e < \dfrac{E}{3} \\[2ex] 1, & \text {if } e \ge \dfrac{E}{3} \end{array}\right. } \end{aligned}$$

The loss function of the conventional PINNs is expressed as follows:

23 $$\begin{aligned} \begin{aligned} \mathscr {L}&={{\lambda }_{f}}{{\mathscr {L}}_{f}}+{{\lambda }_{i}}{{\mathscr {L}}_{IC}}+{{\lambda }_{b}}{{\mathscr {L}}_{BC}} \\&={{\lambda }_{f}}\frac{1}{|{{T}_{f}}|}\sum \limits _{({{x}_{i}},{{t}_{i}})\in {{T}_{f}}}{{{\left| \frac{\partial {{u}^{*}}}{\partial {{t}_{i}}}+{{u}^{*}}\frac{\partial {{u}^{*}}}{\partial {{x}_{i}}}-\frac{0.01}{\pi }\frac{{{\partial }^{2}}{{u}^{*}}}{\partial {{x}_{i}}^{2}} \right| }^{2}}} \\ &+ {{\lambda }_{i}}\frac{1}{|{{T}_{i}}|}{{\sum \limits _{({{x}_{i}},0)\in {{T}_{i}}}{\left| {{u}^{*}}({{x}_{i}},0)+\sin (\pi x) \right| }}^{2}} \\ &+ {{\lambda }_{b}}\frac{1}{|{{T}_{b}}|}{{\sum \limits _{({{x}_{i}},{{t}_{i}})\in {{T}_{b}}}{\left| {{u}^{*}}(\pm 1,{{t}_{i}})\right| }}^{2}} \end{aligned} \end{aligned}$$

To ensure better comparability with WF-PINNs, the loss weights $(\lambda _f, \lambda _i, \lambda _b)$ in PINNs are assigned values of (10, 1, 1) respectively.

**Figure 3:**
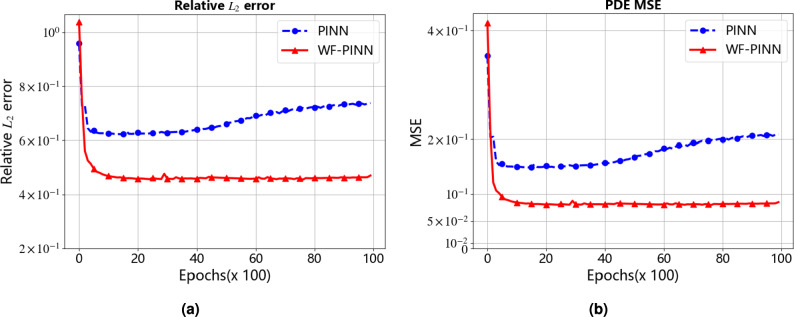
Relative ${{L}_{2}}$-error and MSE During Iteration for the Burgers Equation. (**a**) Relative ${{L}_{2}}$-error of the Burgers Equation. (**b**) Mean Squared Error (MSE) of the Burgers Equation.

Figure 3 presents the evolution of the relative ${{L}_{2}}\text {-}error$ and mean squared error (MSE) during the training processes of both WF-PINNs and standard PINNs. The red curve corresponds to WF-PINNs, while the blue curve represents standard PINNs. In the initial training phase, both models exhibit high MSE and relative ${{L}_{2}}\text {-}error$ values, indicating significant deviations between the predicted and exact solutions. As iterations progress, the relative ${{L}_{2}}\text {-}error$ of WF-PINNs decreases rapidly and consistently, stabilizing at a low level after a certain number of iterations. Its MSE also decreases substantially and eventually remains at a relatively low value, demonstrating the efficient approximation capability of WF-PINNs. In contrast, although the relative ${{L}_{2}}\text {-}error$ and MSE of standard PINNs show some reduction in the early stages, further improvement becomes challenging later on, with even a slight upward trend observed. This reflects the instability of traditional PINNs when dealing with non-smooth solutions. Overall, under identical experimental configurations (e.g., network architecture, optimizer, and data sampling), WF-PINNs exhibits significant advantages in convergence behavior and predictive accuracy, reliably approximating the exact solution and providing superior results for solving the target equations. Standard PINNs, however, underperforms due to its limitations in error control and solution approximation.

The quantitative results presented in Table 2 further validate the aforementioned trends. Upon training up to $8 \times 10^3$ epochs, the relative ${{L}_{2}}\text {-}error$ of WF-PINNs decreased from an initial value of $1.04 \times 10^0$ to $4.62 \times 10^{-1}$ (a reduction exceeding 55%), and the MSE decreased from $4.14 \times 10^{-1}$ to $8.19 \times 10^{-2}$(a reduction of over 80%). Moreover, the error curve remained stable, demonstrating strong convergence stability. In contrast, under the same training duration, the relative ${{L}_{2}}\text {-}error$ of the standard PINNs only decreased from $9.58 \times 10^{-1}$ to $7.21 \times 10^{-1}$(a reduction of less than 25%), and the MSE decreased from $3.53 \times 10^{-1}$ to $2.00 \times 10^{-1}$(a reduction of approximately 43%). Furthermore, after $4 \times 10^3$ epochs, both metrics exhibited a slight increase, highlighting the inherent limitation of traditional PINNs in easily converging to local optima when approximating non-smooth solutions.

From the perspective of intrinsic mechanisms, WF-PINNs optimizes residual point sampling and the loss function, thereby enhancing the enforcement of PDE constraints, boundary conditions, and initial states through interior, boundary, and initial points. This enables the model to focus on error-sensitive regions and avoid undirected weight updates. In contrast, standard PINNs-due to the lack of constraint logic specifically designed for non-smooth solutions-is prone to gradient-related issues in later training stages, hindering convergence or even causing error resurgence.

**Table 2:** Performance metrics of PINNs and WF-PINNs Under the forward problem at different training epochs.

Metric Type	Method	Training Epochs					
		0	2k	4k	6k	8k	10k
Relative ${{L}_{2}}\text {-}error$	PINNs	9.58e-1	6.29e-1	6.40e-1	6.92e-1	7.21e-1	7.37e-1
	**WF-PINNs**	**1.04e0**	**4.58e-1**	**4.59e-1**	**4.58e-1**	**4.62e-1**	**4.70e-1**
MSE	PINNs	3.53e-1	1.52e-1	1.57e-1	1.84e-1	2.00e-1	2.09e-1
	**WF-PINNs**	**4.14e-1**	**8.05e-2**	**8.09e-2**	**8.07e-2**	**8.19e-2**	**8.50e-2**

**Figure 4:**
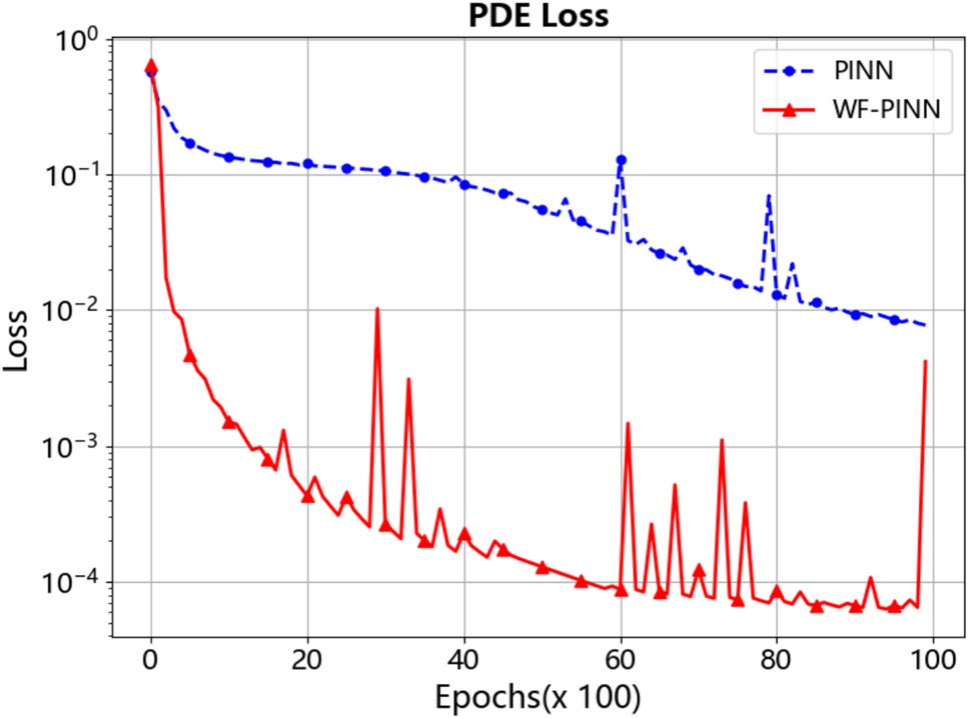
Training loss of WF-PINNs and PINNs during iteration for the burgers equation.

Figure 4 compares the trends of the total training loss values between the proposed WF-PINNs and the standard PINNs throughout the training process. As shown in the figure, the total training losses of both WF-PINNs and PINNs decrease significantly as the number of training epochs increases, indicating effective optimization in both methods. However, throughout the entire training period, the total loss of WF-PINNs remains consistently and markedly lower than that of the standard PINNs at corresponding stages. The loss curve of WF-PINNs exhibits a faster decline rate even in the early phase (within the first 2000-4000 iterations) and continues to maintain a lower loss baseline thereafter. This suggests that incorporating weak solution theory and entropy conditions as physical constraints or regularization mechanisms effectively enhances the efficiency and stability of the optimization process, enabling the model to converge more rapidly to a lower-loss state. These results provide empirical support for the use of additional physical priors-such as entropy conditions-to improve the robustness and accuracy of PINNs.

**Figure 5:**
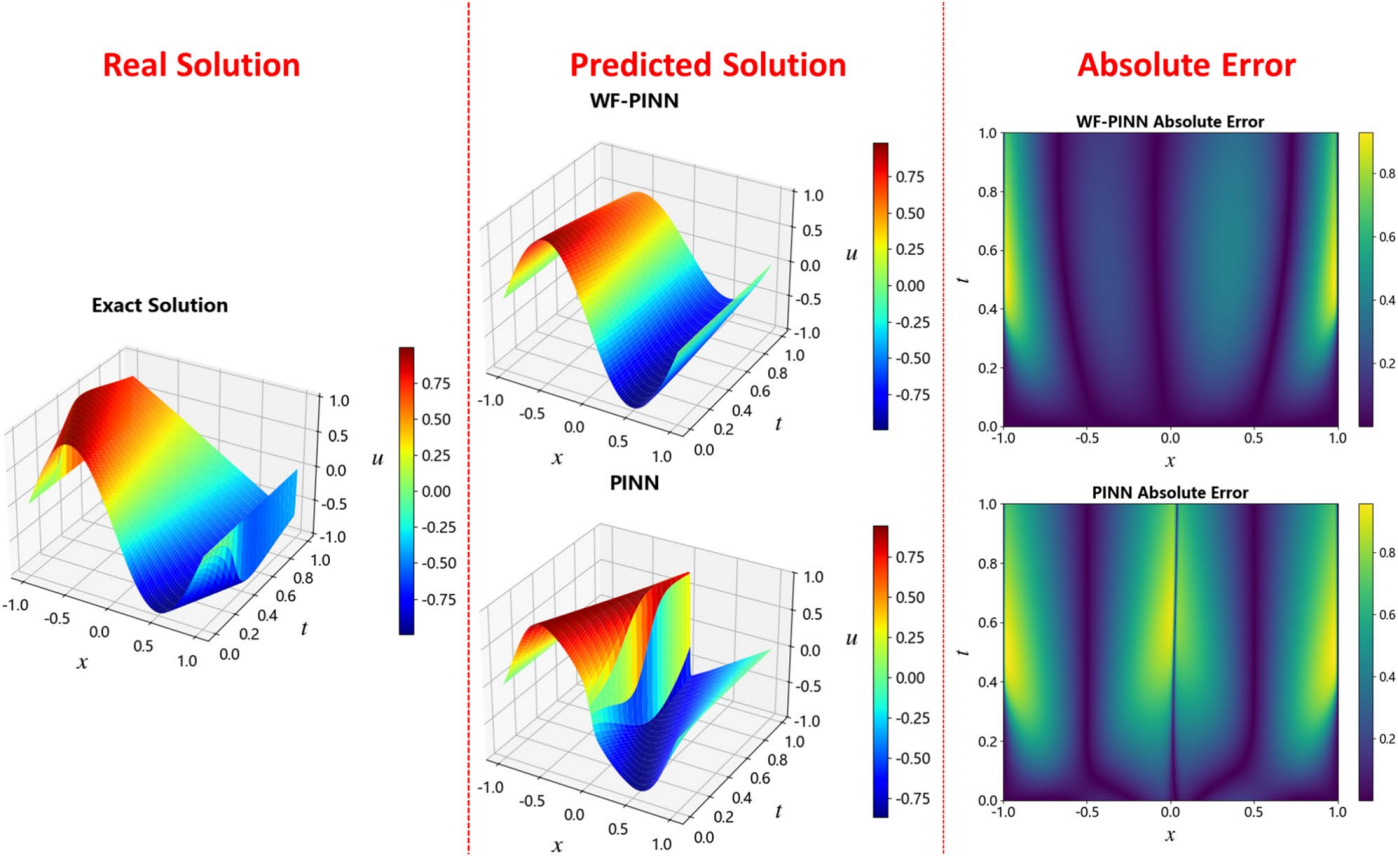
Solution of the burgers equation using different PINN methods.

In research related to physics-informed neural networks (PINNs), where neural networks are constructed to learn solutions of physical equations, it is essential to compare the agreement between the model-predicted solution and the ground truth solution to evaluate model performance. As shown in Fig. 5, the prediction results of WF-PINNs and PINNs, along with the reference ground truth solution, are visualized using 3D surface plots. In these plots, the horizontal axes represent the spatial dimension *x* and the temporal dimension *t*, the vertical axis corresponds to the predicted or true values of the physical quantity *u*, and a color map is employed to further illustrate the distribution of values.

Within the spatio-temporal domain defined by space *x* and time *t*, the solution obtained by WF-PINNs exhibits a continuous and smooth morphology. The color mapping (see colorbar on the right) indicates a gradual transition of the values of *u* from red (high values) to blue (low values), which corresponds closely to the color distribution pattern of the ground truth solution. In comparison, the deviation of the PINNs solution is more pronounced, while the WF-PINNs solution demonstrates closer alignment with the reference solution. The overall morphology of the WF-PINNs solution approximates the distribution of the true physical field both globally and locally. In contrast, the PINNs result shows noticeable macro-scale morphological distortions and local numerical anomalies, whereas errors in the WF-PINNs solution are confined to subtle local features such as surface wrinkles and value transitions. These observations highlight the significant impact of model architecture and constraint design on the quality of the solution.

### Inverse problem of the burgers equation

In this section, we demonstrate the capability of the WF-PINNs framework in addressing inverse problems related to the Burgers equation. To ensure continuity of the research objective and further investigate the inversion problem of the Burgers equation, we revisit the solutions with steep gradients of the Burgers equation as given in Eq. (20), where the initial condition $\phi (x)$ is unknown. Its specific form is shown in Eq. (24):

24 $$\begin{aligned} \begin{aligned}&\frac{\partial u}{\partial t}+u\frac{\partial u}{\partial x}=\frac{0.01}{\pi }\frac{{{\partial }^{2}}u}{\partial {{x}^{2}}},\quad (x,t)\in [-1,1]\times [0,1], \\&u(x,0)=\phi (x), \\&u(-1,t)=u(1,t)=0. \\ \end{aligned} \end{aligned}$$

By utilizing randomly sampled observed data from the reference solution, we apply the WF-PINNs framework to reconstruct the initial condition *u*(*x*, 0) and further infer the full solution *u*(*x*, *t*) of the equation. In the PINN-based framework for initial condition inversion, the observation points consist of a limited number of interior points (non-initial temporal data within the spatiotemporal domain) and a small set of initial observation points (data at the initial time). This configuration represents a common practical setting, particularly in scenarios where experimental data are scarce or measurement costs are high.

Physics-Informed Neural Networks (PINNs) aim to integrate physical principles into neural networks to solve various physical problems. When using PINNs to inversely determine the initial conditions of an equation, the design of the loss function is critical, as it directly affects the model’s ability to accurately recover the true initial condition. In this problem, the boundary conditions and the specific form of the equation are given. This numerical example adopts a dual-network architecture based on a division-of-labor and collaboration strategy, which enables direct inversion of the initial condition. The main network predicts the solution over the entire spatio-temporal domain, denoted as $u^{*}$, while the initial condition network specifically estimates *u*(*x*, 0), outputting the predicted value at the initial time, denoted as $u^{ic*}$.

It is important to note that the first component is the mean squared error related to the residual of the measurement data, which consists of two parts: the initial condition observation loss and the internal observation loss. The former is constructed from a small amount of initial observation data (at $(t=0)$), and the latter is formed from partial internal observation data (avoiding regions prone to shocks). The second component is the initial condition consistency loss, which enforces consistency between the outputs of the two networks at the initial time, ensuring that the output of the initial condition network corresponds to a“slice”of the full spatio-temporal solution at $(t=0)$.

The following are key considerations and common components when designing the loss function. The loss function of WF-PINNs is defined as:

25 $$\begin{aligned} \begin{aligned} \mathscr {L}&= \lambda _w \mathscr {L}_{\text {Weak}} + \lambda _e \mathscr {L}_{\text {Entropy}} + \lambda _b \mathscr {L}_{\text {BC}} + \lambda _m \mathscr {L}_{m} + \lambda _{\text {consist}} \mathscr {L}_{\text {consist}} \\&= \lambda _w \frac{1}{N} \sum _{i=1}^{N} \left| \iint _{\Omega } \left( u_{\theta } \frac{\partial \phi _i}{\partial t} + \frac{u_{\theta }^2}{2} \frac{\partial \phi _i}{\partial x} - u_{\theta } \frac{0.01}{\pi } \frac{\partial u_i}{\partial x} \frac{\partial \phi _i}{\partial x} \right) dxdt \right| ^2 \\ &+ \lambda _e \sum _{j=1}^{M} \left| \max \left( 0, \eta (u_{\theta })_t + \varphi (u_{\theta })_x \right) \right| + \lambda _b \frac{1}{|T_b|} \sum _{(x_i,t_i) \in T_b} \left| u^{*}(\pm 1,t_i) \right| ^2 \\ &+ \lambda _m\frac{1}{|N_{ic}|} \sum _{k=1}^{N_{ic}} \left| u^{ic*}(x_k,0) - u_{\text {obs}_i}(x_k,0) \right| ^2 \\ &+ \lambda _m\frac{1}{|N_{\text {inner}}|} \sum _{l=1}^{N_{\text {inner}}} \left| u^{*}(x_l,0) - u_{\text {obs\_inner}}(x_l,0) \right| ^2 \\ &+ \lambda _{\text {consist}} \frac{1}{|T_i|} \sum _{(x_l,t_l) \in T_i} \left| u^{ic*}(x_l,0) - u^{*}(x_l,0) \right| ^2 \end{aligned} \end{aligned}$$

Regarding the weighting scheme for the five loss components in WF-PINNs, this study assigns values of $\lambda _w = 10$, $\lambda _b = 1$, $\lambda _m = 1$, and $\lambda _{consist} = 10$, This specific configuration is chosen to emphasize the contributions of our core innovations: the weak-form residual ($\mathscr {L}{\text {Weak}}$) and the dual-network consistency ($\mathscr {L}{\text {consist}}$), which are critical for stabilizing the training in the presence of shocks and ensuring synergy between the main and auxiliary networks. The chosen weights were validated through ablation studies, which confirmed that this setup yields superior stability and accuracy compared to a uniform weighting scheme. Similar to the treatment in the forward problem, to prevent the entropy condition from prematurely dominating the optimization direction and leading to convergence to a suboptimal local solution, the training process prioritizes physical residuals in the initial stages and gradually strengthens the entropy constraint in later phases. Accordingly, $\lambda _e$ is dynamically adjusted using a piecewise linear function. The specific variation in the weight can be expressed as follows:

26 $$\begin{aligned} \lambda _e = {\left\{ \begin{array}{ll} \dfrac{3e}{E}, & \text {if } 0 \le e < \dfrac{E}{3} \\[2ex] 1, & \text {if } e \ge \dfrac{E}{3} \end{array}\right. } \end{aligned}$$

The loss function for the conventional PINNs is designed as follows:

27 $$\begin{aligned} \begin{aligned} \mathscr {L}&= \lambda _f \mathscr {L}_f + \lambda _b \mathscr {L}_{BC} + \lambda _m \mathscr {L}_m + \lambda _{\text {consist}} \mathscr {L}_{\text {consist}} \\&= \lambda _f \frac{1}{|T_f|} \sum _{(x_i,t_i) \in T_f} \left| \frac{\partial u^{*}}{\partial t_i} + u^{*} \frac{\partial u^{*}}{\partial x_i} - v \frac{\partial ^2 u^{*}}{\partial x_i^2} \right| ^2 + \lambda _b \frac{1}{|T_b|} \sum _{(x_i,t_i) \in T_b} \left| u^{*}(\pm 1,t_i) \right| ^2 \\&+ \lambda _m\frac{1}{|N_{ic}|} \sum _{k=1}^{N_{ic}} \left| u^{ic*}(x_k,0) - u_{\text {obs}_i}(x_k,0) \right| ^2 \\&+ \lambda _m\frac{1}{|N_{\text {inner}}|} \sum _{l=1}^{N_{\text {inner}}} \left| u^{*}(x_l,0) - u_{\text {obs\_inner}}(x_l,0) \right| ^2 \\&+ \lambda _{\text {consist}}\frac{1}{|T_i|} \sum _{(x_l,t_l) \in T_i} \left| u^{ic*}(x_l,0) - u^{*}(x_l,0) \right| ^2 \end{aligned} \end{aligned}$$

To ensure a more comparable analysis with WF-PINNs, the loss weights of PINNs, namely $\lambda _f$, $\lambda _b$, $\lambda _m$, and $\lambda _{consist}$, are assigned values of 10, 1, 1, and 10, respectively.

**Figure 6:**
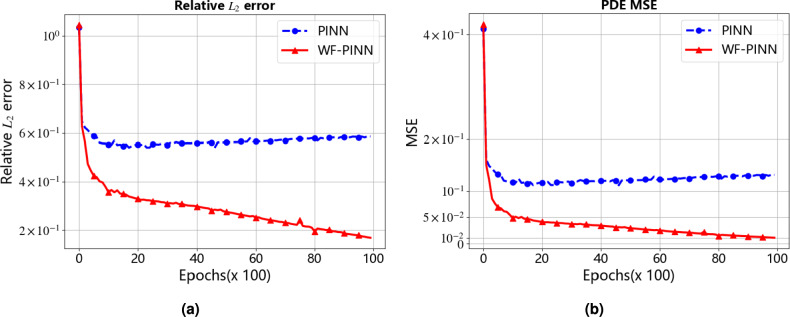
Relative ${{L}_{2}}$-error and MSE during iterative solution of the burgers equation. (**a**) Relative ${{L}_{2}}$-error of the Burgers Equation. (**b**) Mean squared error (MSE) of the Burgers Equation.

First, this section presents two sets of comparative plots of the relative ${{L}_{2}}\text {-}error$ and mean squared error (MSE) for the physics-informed neural networks (PINNs), aiming to evaluate the performance of different PINN methods (WF-PINNs and standard PINNs) in the given task. The horizontal axis represents the training epochs (in units of $\times$100), and the vertical axis indicates the values of the respective metrics. As illustrated in Fig. 6, it can be visually observed that as the training progresses, the proposed WF-PINNs exhibits a significant decrease in both metrics, demonstrating superior performance. In contrast, the MSE of the standard PINNs tends to stabilize in the later stages of training, showing limited convergence, and underperforms compared to WF-PINNs in both evaluation criteria. The results indicate that WF-PINNs achieves better performance compared to the standard PINNs, confirming the practical effectiveness of incorporating strategies such as weak solution theory and entropy conditions.

**Figure 7:**
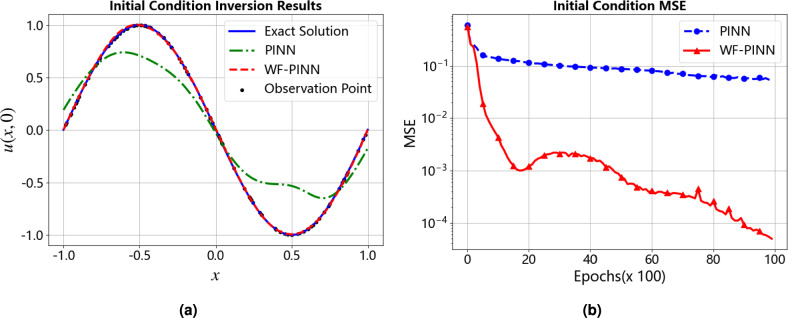
Comparison of initial condition inversion results for the Burgers Equation. (**a**) Comparison of initial condition inversion situations. (**b**) Comparison of MSE situation in initial condition inversion.

Figure 7 presents the inversion results of the initial condition and a comparative analysis of the performance. In the left subfigure, the black dots represent the observation points of the initial condition, which are used to constrain the model learning; the solid line denotes the ground truth initial condition, serving as the evaluation benchmark; and the red dashed line represents the inversion result obtained by WF-PINNs, which exhibits high agreement with the ground truth across most of the spatial domain, demonstrating high inversion accuracy. The right subfigure, which shows the Mean Squared Error (MSE) of the initial condition inversion, further confirms the superior performance of WF-PINNs in numerical prediction. These results indicate that WF-PINNs is more suitable for initial condition inversion tasks, providing valuable guidance for method selection in physics-driven machine learning applications.

**Figure 8:**
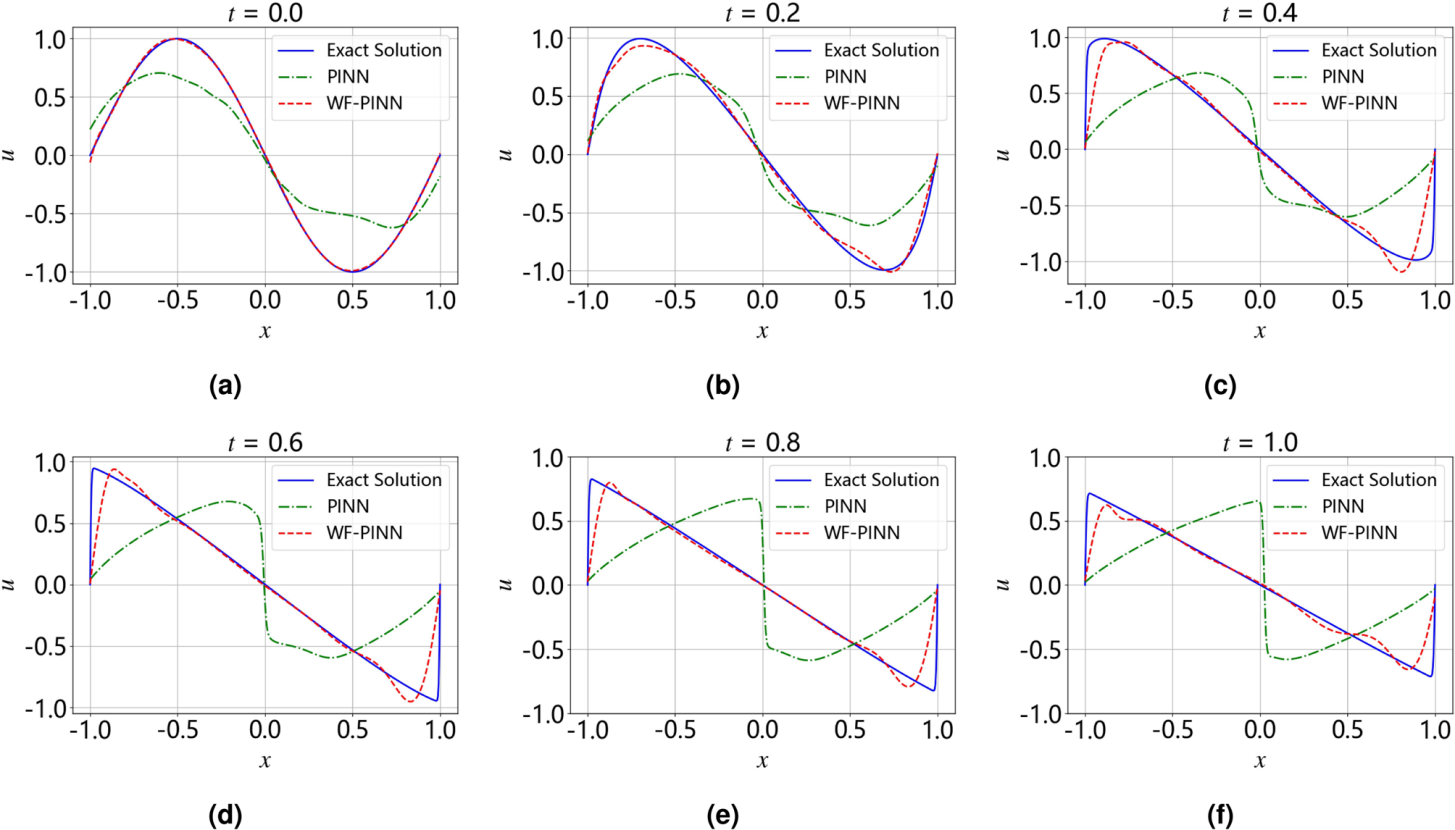
A comparison of the results from the WF-PINNs and PINNs for solving the Burgers’ equation at different time instants. (**a**) $t=0.$ (**b**) $t=0.2.$ (**c**) $t=0.4.$ (**d**) $t=0.6.$ (**e**) $t=0.8.$ (**f**) $t=1.0.$.

Figure 8 compares the inversion results of WF-PINNs and PINNs against the reference solution for the Burgers equation at different time slices. The red dashed line, representing the inversion result of WF-PINNs, shows high agreement with the ground truth in most spatial regions, demonstrating superior inversion accuracy. In contrast, the green dashed line, denoting the result of the strong-form PINNs, exhibits noticeable deviations from the ground truth, indicating relatively inferior inversion performance.

Table 3 compares the mean squared error (MSE) of WF-PINNs and PINNs in reconstructing the spatiotemporal solution $u(x,t)$ and the initial condition $u(x,0)$, revealing significant performance differences between the two methods. WF-PINNs demonstrates superior convergence efficiency and numerical stability in both tasks: the MSE decreased by over 99.9% in reconstructing $u(x,0)$ and by 97.4% in reconstructing $u(x,t)$, with the error curves declining smoothly throughout training. In contrast, PINNs not only exhibited considerably higher final errors but also encountered a noticeable plateau and even a slight error increase in the later training stages, indicating limited convergence performance. These results demonstrate that WF-PINNs, through optimized residual sampling and loss function design, achieves synergistic improvement in both local and global inversion performance, significantly outperforming the conventional PINNs approach and highlighting its reliability and applicability for solving inverse problems involving partial differential equations (PDEs).

**Table 3:** Comparison of MSE for inversion of $u(x,t)$ and $u(x,0)$ at different training epochs.

MSE	Method	Training epochs					
		0	2k	4k	6k	8k	10k
*u*(*x*, *t*)	PINNs	4.11e-1	1.17e-1	1.20e-1	1.23e-1	1.28e-1	1.31e-1
	**WF-PINNs**	**4.20e-1**	**4.70e-2**	**3.40e-2**	**2.45e-2**	**1.46e-2**	**1.09e-2**
*u*(*x*, 0)	PINNs	6.08e-1	1.15e-1	9.37e-2	8.12e-2	6.24e-2	5.52e-2
	**WF-PINNs**	**5.63e-1**	**1.21e-3**	**1.72e-3**	**4.14e-4**	**2.60e-4**	**4.98e-5**

**Figure 9:**
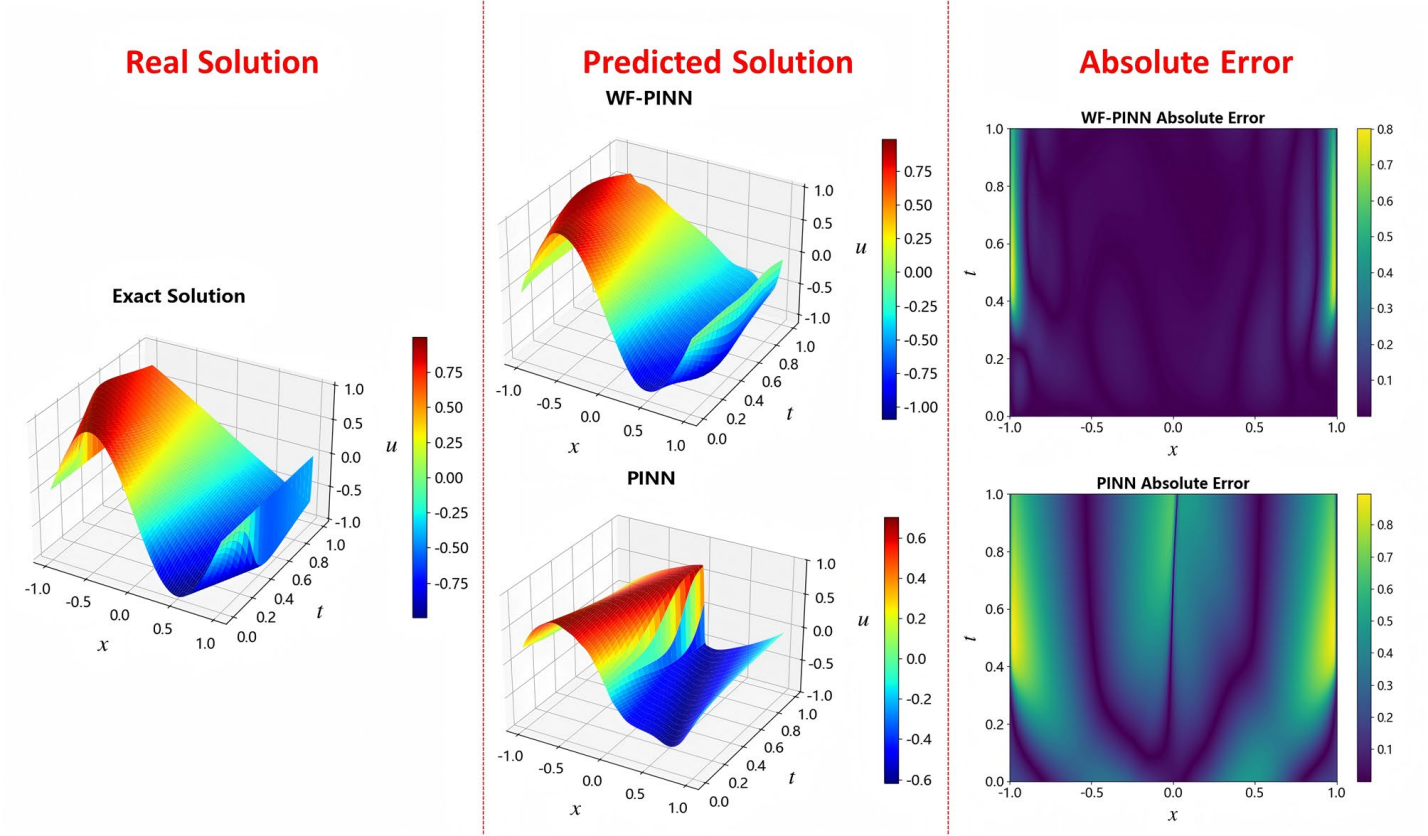
Solutions of the Burgers equation using different PINN methods.

As evidenced by Fig. 9, WF-PINNs demonstrates superior performance in this inversion task, accurately reproducing both the spatiotemporal distribution characteristics and the amplitude range of the ground truth solution. In contrast, the standard strong-form PINNs exhibits significant deviations in its inversion results, indicating a need for architectural improvements (e.g., in the loss function or network depth) or enhanced training strategies. Such comparative results provide an intuitive three-dimensional visual basis for method selection and accuracy validation in physical field inversion and function approximation tasks using physics-informed neural networks.

## Conclusion

In this paper, we provide a detailed introduction to weak solution theory and the resulting WF-PINN framework, which offers a mathematical foundation for Physics-Informed Neural Networks (PINNs) to handle solutions with steep gradients. By replacing pointwise derivative constraints with an integral-form loss function, the neural network achieves more stable convergence toward generalized solutions, thereby enabling more accurate capture of shock waves within the domain and yielding improved results. This approach extends the applicability of PINNs to fields such as fluid mechanics and shock wave-related problems, serving as an essential supplement to strong-form solution methods.

Although the finite difference numerical solution contains inherent discretization errors, it remains a reliable benchmark for neural network methods, as it represents a rigorously validated approach in computational fluid dynamics. The WF-PINN method proposed in this study achieves superior residual control accuracy compared to traditional numerical methods for solving the equations examined herein, demonstrating its effectiveness in addressing nonlinear partial differential equations.

The results further demonstrate that the Weak Form Physics-Informed Neural Networks (WF-PINNs) significantly outperforms conventional PINN methods in both solving the spatiotemporal solution *u*(*x*, *t*) and reconstructing the initial condition *u*(*x*, 0) of the Burgers equation. WF-PINNs effectively overcomes optimization stagnation caused by numerical instabilities in shock regions, demonstrating enhanced applicability and robustness for both forward and inverse problems involving convection-dominated nonlinear partial differential equations.

While the proposed WF-PINN framework demonstrates significant advantages in solving both forward and inverse problems of the Burgers equation, it is not without its limitations, which point to promising avenues for future research. A primary limitation is the computational overhead, as evaluating the weak-form integrals and training the dual-network architecture incur higher computational cost and memory footprint compared to standard PINNs. Future work could explore more efficient numerical integration strategies or adaptive weighting schemes to mitigate this cost. Furthermore, the efficacy and scalability of the current approach for higher-dimensional problems or systems of conservation laws remain to be thoroughly investigated. Generalizing WF-PINNs to such complex scenarios constitutes a critical next step. Moreover, the current understanding of the method’s convergence and stability is primarily based on extensive numerical experimentation; developing a more rigorous theoretical foundation is thus a key challenge. Finally, exploring the integration of data-driven techniques, such as sparse identification of nonlinear dynamics, with the physics-informed constraints of our framework could further enhance its capability for identifying unknown system models.

## Data Availability

The datasets used and analysed during the current study available from the corresponding author on reasonable request.
