# Correction: WF-PINNs: solving forward and inverse problems of burgers equation with steep gradients using weak-form physics-informed neural networks

**DOI:** 10.1038/s41598-026-45558-2

**Published:** 2026-03-30

**Authors:** Xianke Wang, Shichao Yi, Huangliang Gu, Jing Xu, Wenjie Xu

**Affiliations:** 1 School of Science, Jiangsu University of Science and Technology, Zhenjiang, 212003 China; 2 Zhenjiang Jizhi Ship Technology Co., Ltd., Zhenjiang, 212003 China; 3 Yangzijiang Shipbuilding Group, Taizhou, 212299 China; 4 BON BNPP CONSUMER FINANCE CO., LTD, Nanjing, 210002 China; 5 School of Computer, Jiangsu University of Science and Technology, Zhenjiang, 212003 China

Correction to: *Scientific Reports* 10.1038/s41598-025-24427-4, published online 18 November 2025

The original version of this Article contained an error in Equation 2, where the expression “$\frac{\partial u}{\partial t}$” was incorrectly stated as “$\partial u\partial t$”. As a result,

$$\begin{aligned} \int _{0}^{\infty }{\int _{-\infty }^{\infty }{\left({\partial u}{\partial t}+u\frac{\partial u}{\partial x}-v\frac{{{\partial }^{2}}u}{\partial {{x}^{2}}}\right)}}\phi \,dxdt=0. \end{aligned}$$

now reads:

$$\begin{aligned} \int _{0}^{\infty }{\int _{-\infty }^{\infty }{\left(\frac{\partial u}{\partial t}+u\frac{\partial u}{\partial x}-v\frac{{{\partial }^{2}}u}{\partial {{x}^{2}}}\right)}}\phi \,dxdt=0. \end{aligned}$$

Furthermore, Figure 8 contained errors, where panel (a) was duplicated from Figure 4, and where panel (b) and panel (c) were duplicated from Figure 3 panels (a) and (b). The original Figure 8 and accompanying legend appear below.

**Fig. 8:**
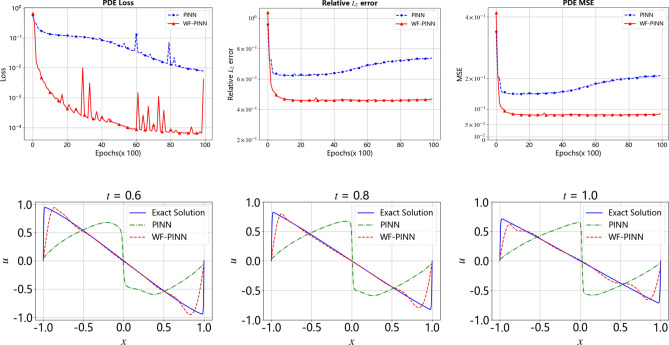
A comparison of the results from the WF-PINNs and PINNs for solving the Burgers’ equation at different time instants. (**a**) $t=0.$ (**b**) $t=0.2.$ (**c**) $t=0.4.$ (**d**) $t=0.6.$ (**e**) $t=0.8.$ (**f**) $t=1.0$.

In addition, Figure 9 was a duplication of Figure 5. The original Figure 9 and accompanying legend appear below.

**Fig. 9:**
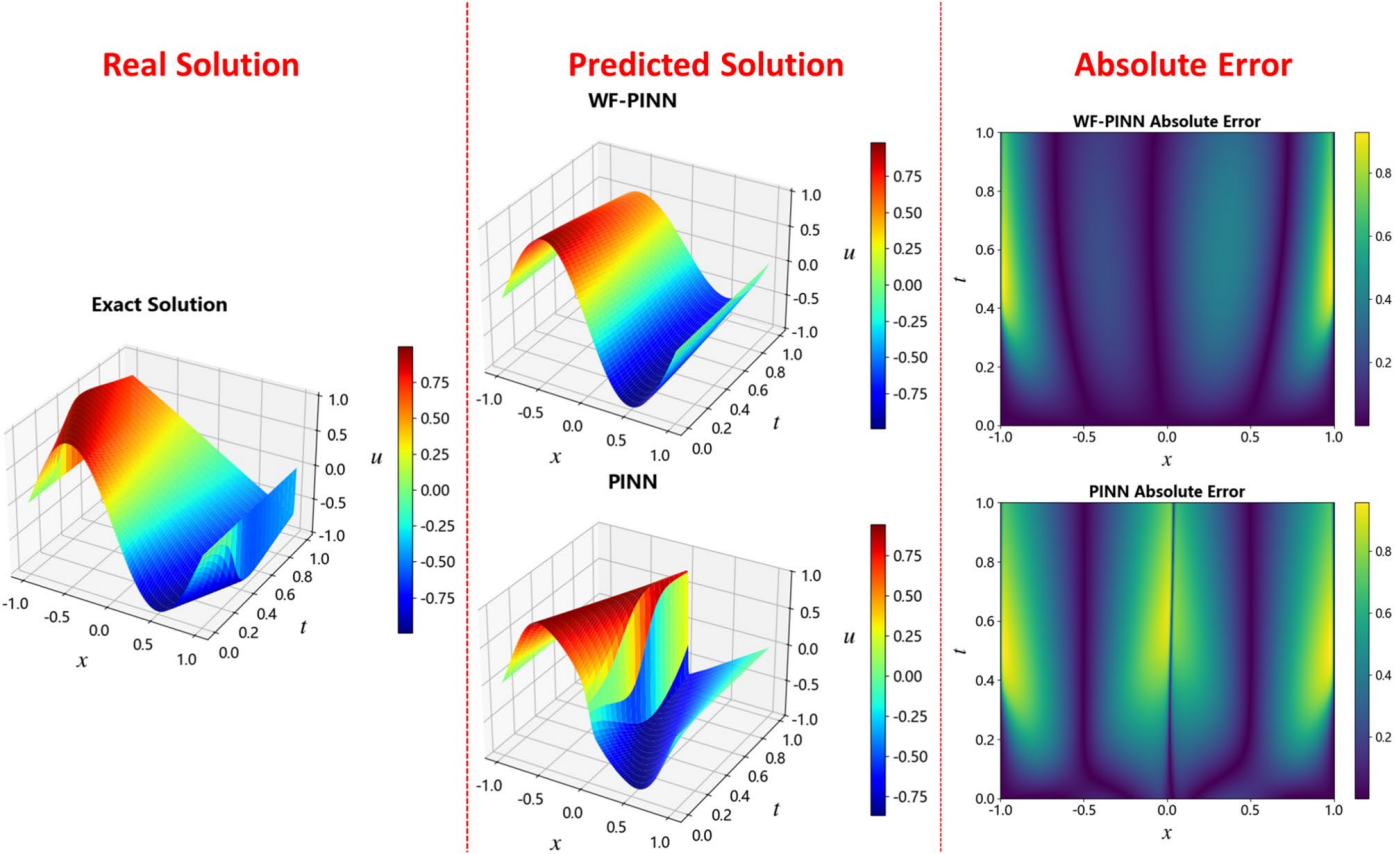
Solutions of the Burgers equation using different PINN methods.

The original Article has been corrected.

